# Cross-Species Extrapolation of Models for Predicting Lead Transfer from Soil to Wheat Grain

**DOI:** 10.1371/journal.pone.0160552

**Published:** 2016-08-12

**Authors:** Ke Liu, Jialong Lv, Yunchao Dai, Hong Zhang, Yingfei Cao

**Affiliations:** College of Natural Resources and Environment, Northwest A&F University, Key Laboratory of Plant Nutrition and the Agri-environment in Northwest China, Ministry of Agriculture, Yangling, China; Sun Yat-Sen University, CHINA

## Abstract

The transfer of Pb from the soil to crops is a serious food hygiene security problem in China because of industrial, agricultural, and historical contamination. In this study, the characteristics of exogenous Pb transfer from 17 Chinese soils to a popular wheat variety (Xiaoyan 22) were investigated. In addition, bioaccumulation prediction models of Pb in grain were obtained based on soil properties. The results of the analysis showed that pH and OC were the most important factors contributing to Pb uptake by wheat grain. Using a cross-species extrapolation approach, the Pb uptake prediction models for cultivar Xiaoyan 22 in different soil Pb levels were satisfactorily applied to six additional non-modeled wheat varieties to develop a prediction model for each variety. Normalization of the bioaccumulation factor (BAF) to specific soil physico-chemistry is essential, because doing so could significantly reduce the intra-species variation of different wheat cultivars in predicted Pb transfer and eliminate the influence of soil properties on ecotoxicity parameters for organisms of interest. Finally, the prediction models were successfully verified against published data (including other wheat varieties and crops) and used to evaluate the ecological risk of Pb for wheat in contaminated agricultural soils.

## Introduction

Pb is a toxin that causes deleterious effects on human health via ingestion of foodstuff grown in Pb-contaminated agricultural soils, especially for children and fetuses [1]. Because of its toxic effects, Pb poisoning is an environmental and public hazard of global proportions [2]. The content of Pb in natural/uncontaminated soils mainly originates from soil parent material, whereas Pb contamination is often a result of anthropogenic activities such as mining, smelting, battery plants, electroplating factories, alloy plants, automobile exhaust, and application of Pb-containing pesticides in agriculture. Consumption of wheat is one of the most important pathways for the entry of Pb into the human body [3], which is of particular significance because wheat is the most commonly consumed dietary staple worldwide, followed by corn and rice [4]. In addition, wheat is an important basic food commodity in northern China. Because of the importance of wheat as a crop and its high susceptibility to Pb contamination, measurement of Pb bioaccumulation in wheat and ecological risk assessment are important issues.

Pb in the soil is generally present in a divalent state within the topsoil (0–15 cm) and shows little ability to migrate deeper. In general, variation in Pb uptake and distribution in plants is dependent on species and soil factors [1, 5]. The average Pb content in cereal samples (n = 59, 0.0564 mg·kg^-1^) from north-eastern China was dramatically higher than that of pulses (n = 34, 0.033 mg·kg^-1^) [2]. In addition, significant differences were observed in the accumulation of Pb among 30 Chinese wheat cultivars, suggesting that genotype was an important factor influencing Pb uptake within the species [6]. Previous studies confirmed that concentrations of heavy metals, including Pb, in wheat grain were significantly associated with soil properties, including pH, organic matter content, cation exchange capacity (CEC), and clay content [7]. Among these factors, pH was found to play a predominant role in affecting the uptake of Pb by plants, because co-precipitation of heavy metal compounds with calcium or magnesium cations reduced the solubility of metal cations at pH > 6, whereas lower soil pH (<6) increased the migratory capacity of cations in soil solution, greatly facilitating absorption of metals by plants. Soil organic matter was also recognized as an important factor influencing Pb uptake by plants. For example, humic acid can form a stable or water-insoluble complex with Pb that inhibits Pb transfer in soil-plant systems. In addition, atmospheric deposition and other routes of surface contamination during harvest and storage result in pollution of wheat grain by Pb [8]. To investigate heavy metal transfer in plant-soil systems, predictive models that account for the substantial influence of soil type on the availability and transfer of metals must be developed [9, 10]. Significant effort has been focused on developing models to predict the extent of Pb transfer from the soil to edible parts of vegetables; however, few attempts have been made to predict Pb uptake and accumulation by cereals, such as wheat, rice, and corn. Currently used prediction models of predicting Pb uptake by grains are restricted to specific contaminated sites and a narrow range of soil properties.

In recent years, species sensitivity distribution (SSD) methodology based on cross-species extrapolation methodology has been applied extensively for ecological risk assessments of hazardous materials, which has led to the establishment of environmental quality standards [11, 12]. Ideally, individual species-specific bioavailability models should be developed for every species of interest. However, acquiring sufficient toxicological data to allow the development of Pb uptake prediction models tailored for every species of interest is difficult due to the high cost and length of time necessary to perform the required studies; therefore, extrapolation approaches are a pragmatic and effective method of predicting ecotoxicity for a wide range of organisms. Cross-species extrapolation has primarily been applied in aquatic environments via the biotic ligand models (BLMs) approach. For example, the Ni prediction BLM developed using the cladocera *Daphnia magna* and *Ceriodaphnia dubia* was applied to three other invertebrates, the snail *Lymnaea stagnalis*, the insect *Chironomus tentans*, and the rotifer *Brachionus calyciflorus*, based on predicted EC_50_ (median effect concentration) values [13]. Cross-species extrapolation of metal bioavailability models based upon EC_50_ or LC_50_ (median lethal concentration) values was further supported by the application of the Ni bioavailability model for *rainbow trout* to the *fathead minnow* in synthetic and natural waters [14], application of the Ni accumulation model for *Daphnia magna* to *Ceriodaphnia dubia* [15], application of the Ni toxicity model for *Ceriodaphnia dubia* to other daphnids [16], and application of the Ni uptake prediction model for green microalgae *Pseudokirchneriella subcapitata* to other green algae species [17]. However, few studies have been performed regarding cross-species extrapolation of metal uptake and accumulation models in terrestrial ecosystems.

In the present study, we aimed to: (1) identify the major factors influencing Pb transfer and develop bioaccumulation factor (BAF)-based predictive models of Pb uptake for a wide range of Chinese wheat-producing soils with different soil Pb levels; (2) estimate the possibility of applying the developed predictive models for cultivar Xiaoyan 22 to six additional non-modeled wheat varieties; and (3) assess the reduction of intra-species variability in Pb uptake for non-modeled crops after normalization of BAF.

## Materials and Methods

### Soil samples

Soil samples were collected from wheat-producing locations in 17 Chinese provinces containing a wide range of soil properties (Table 1). No soil samples were collected from national parks, private land, or protected land, so obtaining special permission for each location was unnecessary. All soil samples from the upper layer (0–20 cm) were air-dried, ground, and passed through a 2-mm sieve as preparation for the subsequent analysis. Soil properties were determined by routine methods [18]. Finally, we confirmed that our studies did not involve any endangered or protected plant species in China. No wheat varieties tested in this study were under first- or second- grade state protection, nor were any varieties listed in the Inventory of Rare and Endangered Plants of China (http://jky.qzedu.cn/zhsj/zxzw/zxzwzy.htm) or the Key Protected Inventory of Wild Plants of China (http://zrbhq.forestry.gov.cn/uploadfile/zrbh/2010-10/file/2010-10-14-bb296addeaa047798d6b6c476aaa1da9.doc). The wheat species were used only for scientific research as permitted by the Ministry of Agriculture of the People's Republic of China.

**Table 1:** Basic physicochemical properties of soils from 17 sampling locations.

Soil number	Location	pH	OC	CEC	Clay (%)	Background Pb
			(g·kg^-1^)	(cmol·kg^-1^)		(mg·kg^-1^)
1	Qiyang, Hunan	4.9	9.00	10.85	42.91	28.74
2	Beibei, Chongqing	5.74	10.14	21.34	24.96	38.46
3	Shenyang, Liaoning	5.74	14.99	12.19	17.32	31.60
4	Kunming, Yunnan	5.92	19.87	11.10	27.52	42.51
5	Yingtan, Jiangxi	6.01	6.78	8.70	36.51	34.75
6	Hefei, Anhui	6.25	11.62	19.08	16.84	30.17
7	Hailun, Heilongjiang	6.27	20.70	28.59	19.33	44.79
8	Gongzhuling, Jilin	6.82	19.05	31.11	30.18	44.73
9	Changshu, Jiangsu	6.93	27.66	26.20	45.94	37.48
10	Yangling, Shaanxi	7.90	9.56	22.37	26.01	37.40
11	Zhengzhou, Henan	8.07	10.32	16.01	18.18	36.12
12	Urumqi, Xinjiang	8.12	11.27	25.25	9.57	40.87
13	Taigu, Shanxi	8.24	13.44	16.80	17.74	38.58
14	Tianjin	8.29	12.77	24.67	7.59	39.75
15	Zhangye, Gansu	8.37	11.18	11.23	6.66	36.86
16	Dezhou, Shandong	8.65	6.87	13.09	17.11	36.02
17	Baotou, Inner Mongolia	8.8	9.45	11.61	10.51	40.80

### Pot experiment design

#### Pb enrichment in different soils

A pot-culture experiment was carried out in a greenhouse in Yangling District, Shaanxi Province, China using wheat cultivar Xiaoyan 22, which served as the main food for local residents. Xiaoyan 22 wheat was grown in the 17 soil samples described above. Three treatments were performed in triplicate based on the Pb limits of the Chinese Secondary Environment Quality Standards for Soils (GB 15618–1995). For the control treatment (CK), no Pb was added to the soil. For the low Pb treatment, 125, 150, or 175 mg·kg^-1^ Pb was added to soils with pH <6.5, 6.5–7.5, and >7.5, respectively. For the high Pb treatment, 250, 300, or 350 mg·kg^-1^ Pb was added to acidic, neutral or alkaline soils, respectively. Soil samples (8 kg each) were placed in plastic pots with a rim diameter of 35 cm and a height of 30 cm to facilitate normal wheat growth and preclude the possibility of nutrient deficiency. The basic fertilizer contained 0.15 g·kg^-1^ N (in the form of CO(NH_2_)_2_), 0.05 g·kg^-1^ P (in the form of Ca(H_2_PO_4_)_2_), and 0.1 g·kg^-1^ K (in the form of K_2_SO_4_) mixed thoroughly with exogenous Pb (in the form of Pb(NO_3_)_2_) in solution. The fertilizer had no significant influence on the Pb uptake of wheat. Then the soil samples were left to equilibrate for three months to allow natural equilibration of the various compounds in the soil, after which wheat plants were grown in untreated and treated soils. The growing period was approximately 210 days (late October to late May). Soil moisture content was kept at approximately 80% of the water holding capacity of the soil using deionized water (ASTK CSR-1-10II pure water, China) to preclude the possibility of adding Pb during the growing period.

#### Additional wheat varieties

Two experimental treatments were performed (three replicates each) using soils from Shaanxi (pH >7.5) and Jiangxi Provinces (pH <6.5) with a large difference in pH. For the control seedlings, no Pb was added to the soil. Pb was added to the Jiangxi soil samples at a concentration of 250 mg·kg^-1^, whereas 350 mg·kg^-1^ Pb was added to the Shaanxi soil samples. After three months of soil equilibration, wheat varieties Hengmai 5229, Jimai 22, Shixin 618, Wanmai 52, Xumai 30, and Zhengmai 9023, the main sources of stable flour in China at present, were planted in each type of soil. The procedures for growing the seedlings, as well as the planting time, were the same as those used in the enrichment experiment for different types of soil.

#### Soil and plant analyses

Soil samples were air dried and passed through a 0.149-mm nylon mesh sieve. Total Pb was measured by a microwave-assisted digestion method in an oven (HNO_3_ (Guaranteed reagent–GR, Kelong, China)-HF (GR, Kemio, China)). The Pb concentrations in the digestion solutions were determined via flame atomic absorption spectrometry (FAAS; Hitachi z-2000, Japan). Dehulled wheat grain was dried at 100°C in an oven. The heating process was repeated until the weight remained constant, after which the plant samples were digested with HNO_3_-H_2_O_2_ (GR, Kelong, China) in a high pressure, sealed microwave digestion oven in accordance with the Determination of Lead in Foods standard (GB/T 5009.15–2003). After the digestion and evaporation procedure, the Pb concentration in each sample was measured via FAAS and graphite furnace atomic absorption spectrometry (GFAAS; Hitachi z-2000, Japan). Quality control was carried out using certified wheat reference material (GBW 10011, China) and soil reference material (Gss-7, Gss-8; China) obtained from the National Research Center for Standard Materials in China (S1 Table), which were tested with the other plant and soil samples. Before the analysis, all glassware and Teflon tubes were soaked in an acid bath (25% HNO_3_) for 12 hours.

### Data analysis

#### Interpretation of BAF

The BAF was calculated as the ratio of the Pb concentration in plant tissue to that in the soil, as follows [19]:

$$BAF=C_{wheat}/C_{soil}$$ (1)

where *C*_*wheat*_ is the Pb concentration in the wheat grain and *C*_*soil*_ is the Pb concentration in the soil.

#### Pb-BAF prediction model in wheat grain

Soil properties are incorporated into models used for metal exposure assessment in crops because soil characteristics have an important impact on uptake of heavy metals by plants [10]. Therefore, multiple stepwise regression was carried out to establish the relationship between soil properties and Pb-BAF, leading to the development of an empirical prediction model for BAF based upon exogenous soil Pb, as follows:

LogBAF=a×pH+b×LogOC+k (2)

where LogBAF and LogOC are the common logarithm values of the BAF and OC content (g·kg^-1^), respectively. The slopes of soil property parameters (*a* and *b*) reflected the relationships between soil properties and Pb accumulation in wheat grain, whereas the intercept *k* was the intrinsic sensitivity, which indicated the variation in Pb uptake among the wheat cultivars.

#### Cross-species extrapolation

For the cross-species extrapolation, it was assumed that the relationships between Pb accumulation in wheat grain (BAF) and soil factors (pH, OC, etc.) were constant among related species [20]. In other words, the slopes of the soil parameters (*a* and *b*) were taken as constant in the prediction models for the six non-modeled wheat cultivars; only the intrinsic sensitivities (*k*) of different wheat varieties to metal were changed during the extrapolation. To minimize the error sum of squares between the predicted and measured BAF values for a single variety in various soils, calculated as $\sum_{i=1}^{n}{\left(measured\;BAF–predicted\;BAF\right)}^{2}$, the intercepts (*k*) of the prediction models for the six non-modeled wheat varieties were obtained using Excel Solver for linear optimization [21]. For this article, the prediction models of related varieties were extrapolated from that of cultivar Xiaoyan 22.

#### Analysis of intra-species variability

The BAF values of the six non-modeled wheat varieties in Jiangxi and Shaanxi soils were normalized to specific soil scenarios based on physico-chemical properties through the calculated model of cultivar Xiaoyan 22. Regardless of whether the Pb-BAF of the related varieties normalized, the intra-species variability of each wheat cultivar was calculated as follows [22]:

$$f=\sqrt{\frac{\sum_{i=1}^{n}{\left({BAF}_{i}–\bar{BAF}\right)}^{2}}{{\left(n–1)\times (\bar{BAF}\right)}^{2}}}$$ (3)

where *f* is the intra-species variability, *BAF*_*i*_ is the BAF of the designated wheat species after normalization to the *i*-th soil condition, *n* is the number of soil conditions for the designated wheat species; and $\bar{BAF}$ is the mean value of n BAFs. Theoretically, the Pb-BAF of a single wheat species in different soils normalized to a given soil condition should be equal. Therefore, normalization to reduce intra-species variability in predicted Pb transfer can eliminate the influences of soil properties from different sources on calculations of Pb accumulation in wheat grain.

## Results and Discussion

### Pb bioaccumulation in wheat grain from different soils and cultivars

**Fig 1:**
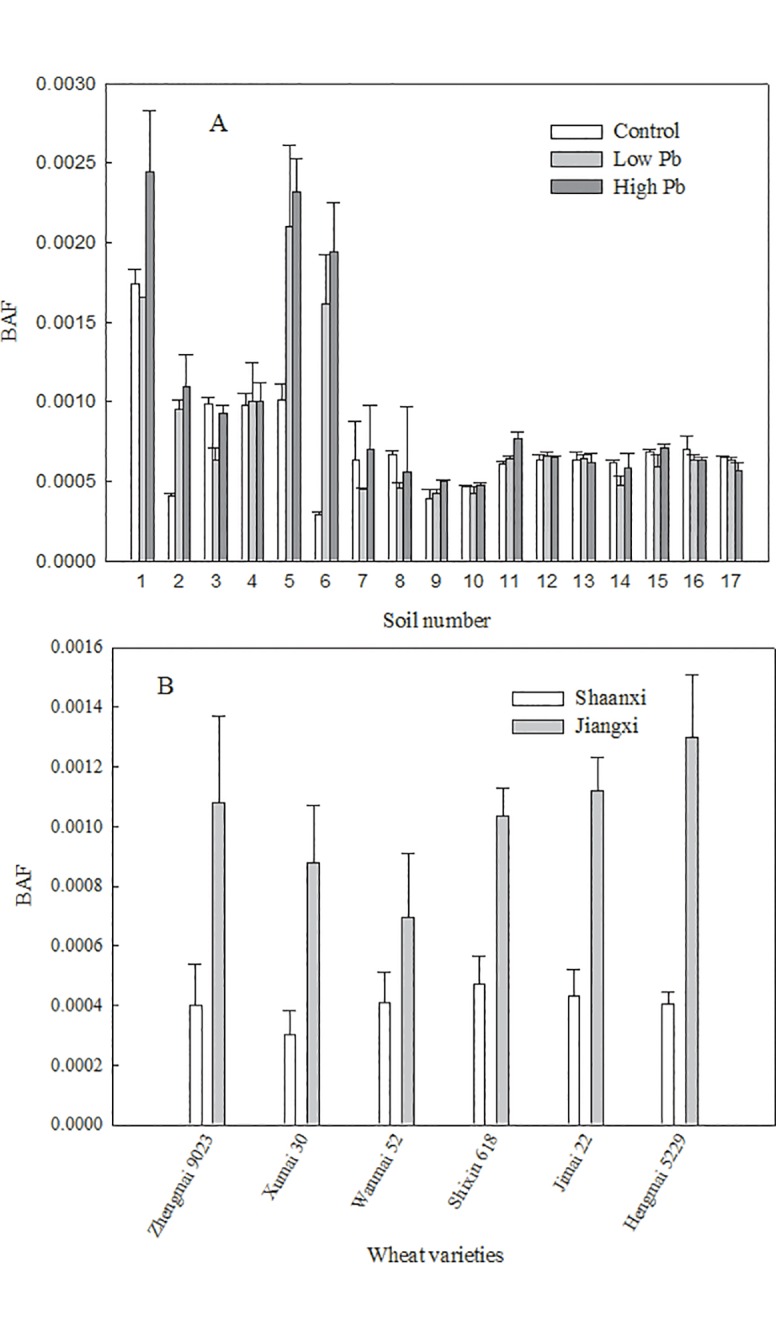
Effects of soil type (A) and wheat variety (B) on the bioaccumulation factor of Pb.

The BAF values for wheat plants grown in soil samples from 17 Chinese provinces varied widely from 0.0003 to 0.0017 (CK), 0.0004 to 0.0021 (low Pb), and 0.0005 to 0.0024 (high Pb) (Fig 1A). Our results agreed with previous studies showing that most crops absorb less than 0.3% of Pb contained in soil. For the Pb treatment, there were remarkable differences in the BAFs of plants grown in acidic and alkaline soils. The BAFs of soils 1, 5, and 6 were higher than those of the other soils. The low BAF of soils 2, 3, and 4 might be attributed to high OC content. The overall trend of the 17 BAF values of the control plants was similar to that of the Pb-treated plants. There were no significant differences in BAF among the control and Pb-treated plants grown in alkaline soil, which indicated that Pb accumulation in wheat grain might not vary much under the stress of Pb pollution, especially in alkaline soil. While pH had a significant influence on Pb absorption by wheat grain, other physicochemical soil properties, including OC and CEC, may also influence Pb accumulation in wheat grain. In a previous study of Pb accumulation by wheat grown in contaminated soil from an oasis in north-western China (pH 8.16), the BAF of the grain (0.0004) was close to the average BAF in alkaline soil measured in the present study (0.0006) when the soil Pb concentration (342 mg·kg^-1^) approached the amount of exogenous Pb added in the present study (350 mg·kg^-1^) [23]. The Pb-BAF of wheat grain across Britain from 1982–2000 [8] (n = 588; soil Pb content, 15–772 mg·kg^-1^; plant Pb content, <0.02–1.63 mg·kg^-1^) was about 0.00036–0.029, which was slightly greater than that measured in the present study. In a recent literature review, it was suggested that the BAF of Chinese wheat grain was approximately 0.00018–0.014 [24].

A previous study indicated that the Pb-BAF of crops was dependent on plant species, in addition to soil properties and experimental setup [25]. The present study confirmed significant differences in Pb-BAF among six wheat cultivars grown in alkaline and acidic soils. The Pb-BAF of wheat cultivars grown in Jiangxi soil ranged from 0.0003 (Xumai 30) to 0.0005 (Shixin 618), whereas that of wheat cultivars grown in Shaanxi soil ranged from 0.0007 (Wanmai 52) to 0.0013 (Hengmai 5229) (Fig 1B). The BAF value for each cultivar in acidic soil was higher than the corresponding BAF in alkaline soil. There was slight variation in the BAF values of the six wheat cultivars in alkaline soil, but the corresponding BAFs varied widely in acidic soil. These results show that alkaline soil could be used to prevent wheat plants from accumulating soil Pb. In addition, these findings demonstrate that, among the six tested cultivars, Wanmai 52 absorbed the least Pb from the soil, where Hengmai 5229 absorbed the most Pb from the soil into the wheat grain; this knowledge could be useful for screening Pb-resistant cultivars. In a previous pot experiment conducted to assess the transfer of Pb to wheat grain grown in soil containing sewage sludge (soil Pb concentration, 75.5 mg·kg^-1^), the BAF range of the four tested varieties was 0.264–0.299, which was higher than that of plants grown in contaminated soil in the present study [26].

### Prediction model of Pb-BAF in wheat grain

BAFs were applied to characterize the enrichment of heavy metals within the food chain based on recommendations for the processes of assessing environmental risk and developing soil environmental quality standards [27]. Multiple linear regression is commonly used to generate models for predicting heavy metal concentrations in soil and plants based on physical and chemical characteristics. Log-transformed data was used to improve the linear fitting of the predictive equation based on previous studies. For example, the *R*^2^ of predictions for Cd transfer from the soil to spinach was increased from 0.534 to 0.878 after the data were log-transformed [28].

A stepwise linear regression was performed to establish the relationships between soil parameters, including pH, OC, CEC, and clay content, and the Pb-BAFs of grain grown in 17 types of soil using Eq 2. We chose these four soil properties because some technical documents (e.g., Guidance for Developing Ecological Soil Screening Levels by U.S. Environmental Protection Agency) reported that they were the most important factors affecting the bioavailability of heavy metals. For examples, some authors predicted Cd, Cu, Pb, and Zn concentrations in wheat grain and leaves from soil concentrations using these properties [29]. The Pb uptake of roots was determined by soil properties, especially soil pH and organic matter, in addition to total soil Pb content [30, 31]. The empirical predictive model of BAF for wheat grain in different Pb treatments was established based on cultivar Xiaoyan 22 (Table 2):

**Table 2:** Prediction models for the different Pb treatments.

Model NO.	Treatment	Prediction models	R^2^	*p*	n
Model 1	Low Pb	Log BAF = -0.121pH-0.808Log OC-1.399	0.69	<0.001	17
Model 2	High Pb	Log BAF = -0.151pH-0.740Log OC-1.194	0.80	<0.001	17

**Fig 2:**
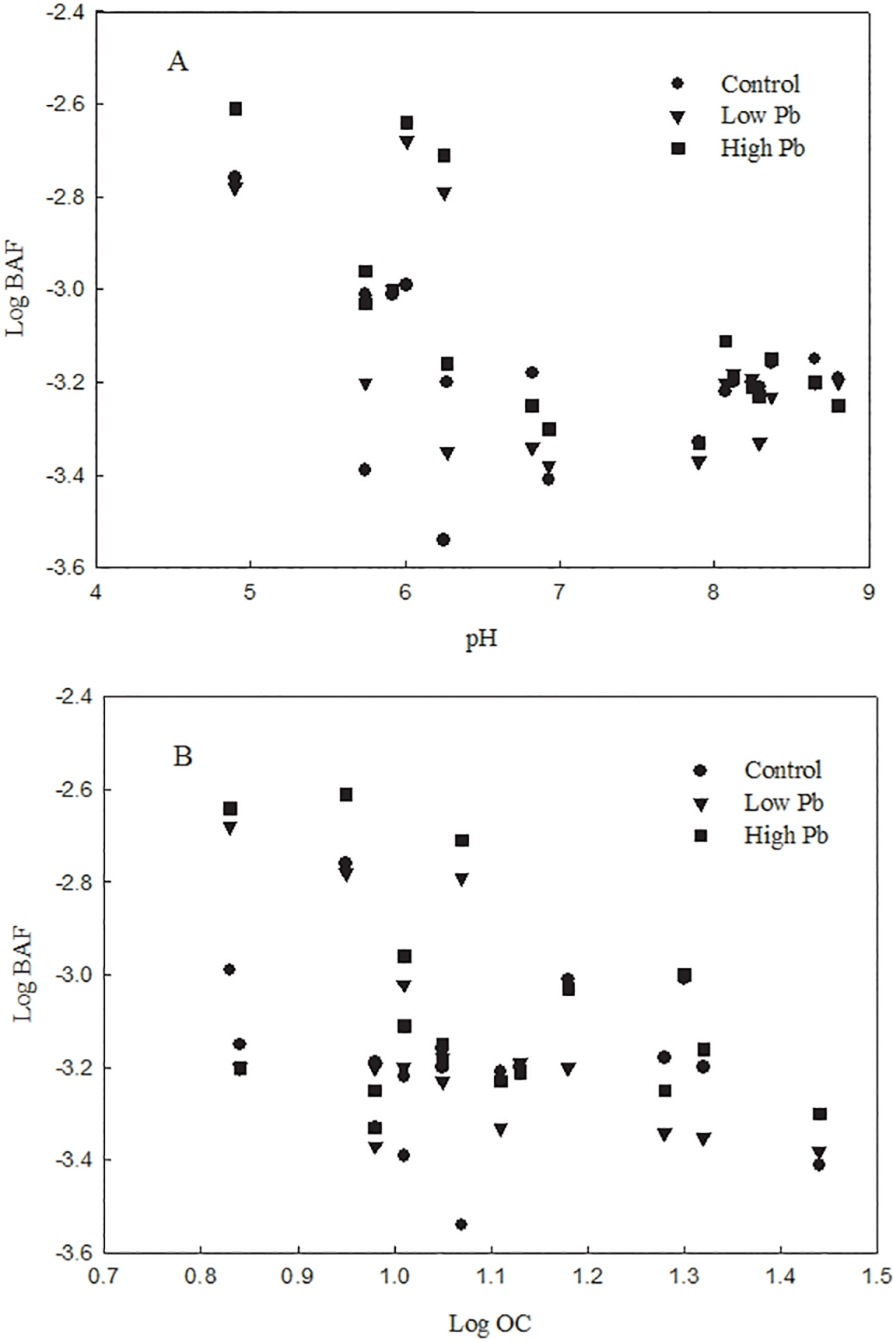
Relationship between Pb accumulation in wheat grain and soil properties (A, pH; B, LogOC).

As shown in Table 2, the results of the Pb treatments (low and high) were in agreement with the analysis of Pb bioaccumulation in different soils (section 3.1), indicating that a highly significant interaction existed between BAF and soil pH, as well as between BAF and OC. The pH value influences the partitioning of heavy metals between the soil and water phases. In addition, complexation of soil organic matter can play a key role in immobilization of metal ions in soils, reducing phytotoxicity. The Log BAF of the grain markedly declined as pH or OC was increased in the control and Pb treatments (Fig 2A and 2B). Our analysis showed that the workable ranges of pH and OC for the 17 tested soil samples varied widely (pH, 4.9–8.8; OC, 6.78–27.66 g·kg^-1^) and were broad enough to manage the variation of the two predictors in the regression models. Spring wheat grown in soil near a base for mining and smelting of non-ferrous metals in China showed that the most significant influence on BAF and Pb uptake in wheat grain was the total soil Pb concentration [7]. Soil pH is an important influence on the availability and mobility of metals, including Pb, in soil-plant systems [32]. Therefore, farmers dealing with heavy metal contamination can increase the pH of the soil and grow cultivars that accumulate less metal [33]. In addition, soil organic matter content was recognized as the controlling factor influencing the spatial distribution of Pb in the soil of a roadside rice-wheat rotation system in eastern China [30]. In general, our finding that pH and OC were the primary factors influencing absorption of Pb by wheat plants in 17 Chinese soils was consistent with previously published data.

### Cross-species extrapolation for non-modeled wheat cultivars

Toxicological parameters of terrestrial plants applied in prediction models have mainly consisted of acute toxicological data (e.g., EC_50_ or LC_50_ values in terms of inhibition of root elongation or seed germination) because of the convenience of using such data and the speed of data collection. For examples, a model of Ni toxicity threshold values based on EC_50_ values developed by assessing barley root length in 16 European soils showed that soil CEC was the primary soil factor affecting Ni uptake [34]. In addition, an EC_50_-based model for predicting Cu uptake by barley was applied to predict Cu toxicity in rice, onion, radish, mustard, and cabbage [20]. However, long-term studies including data such as BAF, which seem to better reflect the toxic effects of heavy metals in the terrestrial environment, are lacking.

**Fig 3:**
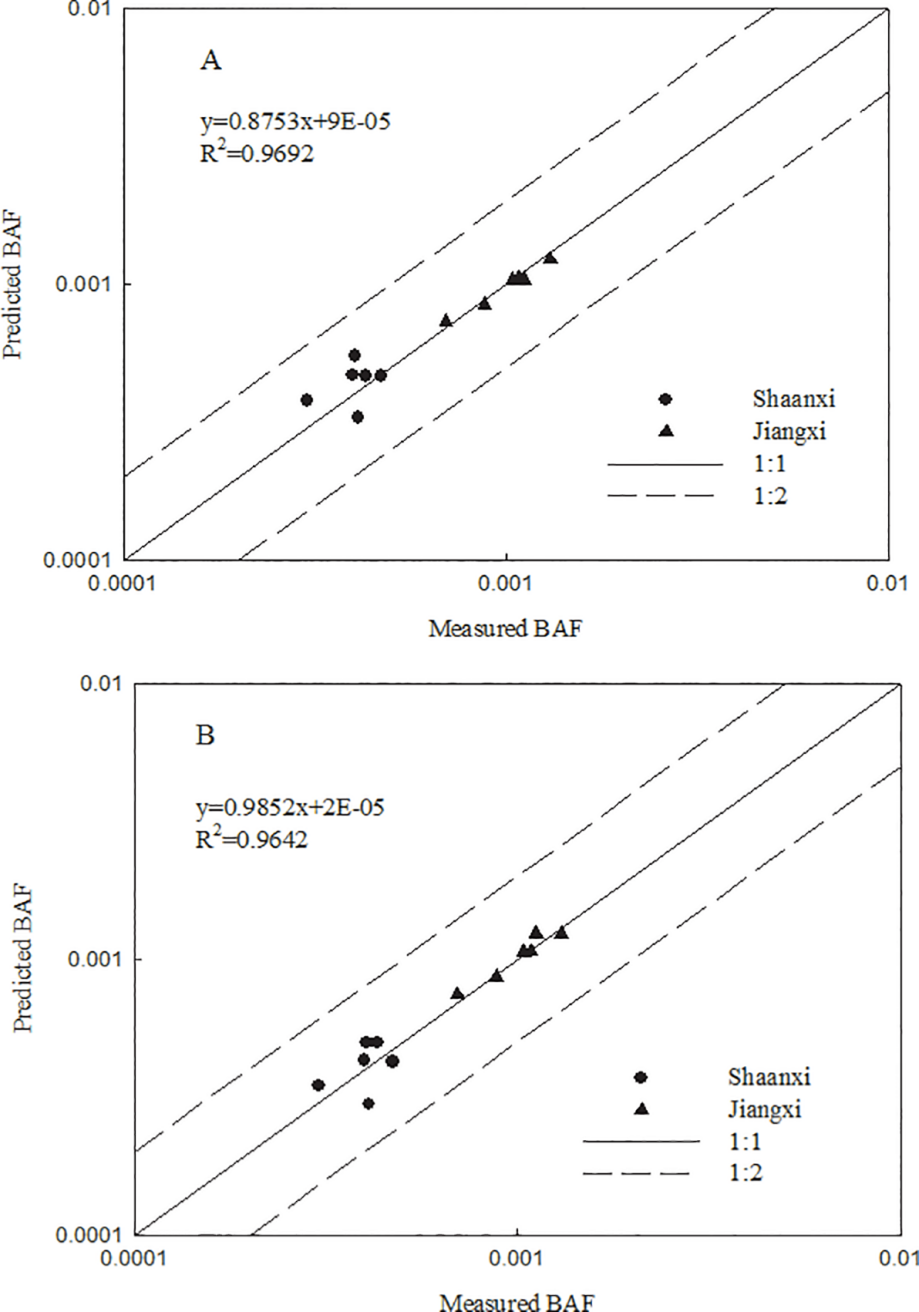
Relationship between measured and predicted BAF for six wheat varieties in Shaanxi and Jiangxi soils (the solid line indicates a perfect correlation; the dashed lines represent a 2-fold prediction interval; A, model 1; B, model 2).

Previous studies have confirmed the utility of cross-species extrapolation of heavy metal toxicity prediction models among different plants [21]. Because there were significant differences in the grain Pb-BAF among six tested wheat cultivars in soils from Shaanxi and Jiangxi provinces, prediction models for the six non-modeled wheat varieties were derived from that developed for cultivar Xiaoyan 22. We assumed that the Pb toxicity of all non-modeled varieties was affected by soil properties (pH and OC) to the same degree; in other words, the slopes of the soil properties in prediction models 1 and 2 (Table 2; model 1: a = -0.121, b = -0.808; model 2: a = -0.151, b = -0.74) were constant. Therefore, the altered *k* for each non-modeled cultivar represented the inherent sensitivity of each variety to Pb accumulation. To minimum the error between predicted BAF and measured BAF for the six non-modeled wheat varieties in soils from Shaanxi and Jiangxi provinces, a set of *k* values for the prediction models in soils with different Pb levels was obtained by linear optimization. The *k* range for the six assessed wheat varieties was from -1.74 to -1.51 and from -1.38 to -1.61 for the low and high Pb treatment, respectively (Table 3). The *k* value of each wheat cultivar in the low Pb soil was smaller than that of each cultivar in the high Pb soil and similar to the *k* value included in the prediction models developed for Xiaoyan 22 (*k* = -1.399, -1.194). In a previous study of Pb uptake by corn [22], extrapolated predictive models for Pb transfer from cultivar Zhengdan 958 (*k* = -1.894, -1.806) to six corn varieties revealed that *k* ranged from -1.82 to -2.30 and from -1.68 to -1.86 in soils with low and high Pb levels, respectively, which was similar to the range of *k* values revealed using our soil Pb concentration set. Therefore, similar to our findings in wheat, corn varieties also showed a limited difference in the range of *k* when exposed to an equal Pb concentration. The accuracy of the Pb accumulation prediction model developed for each of the six non-modeled wheat varieties was estimated by comparing the measured BAF with the predicted BAF. The measured BAF was close to the predicted BAF for each of the six non-modeled cultivars in soils from Shaanxi and Jiangxi provinces. Moreover, all of the actual BAF values were within 2-fold of the predicted BAF values for models 1 and 2, showing that the two models were accurately predicted Pb-BAF of grain in soils with different Pb levels (R^2^ > 0.96). The predictive accuracy of model 1 was slightly better than that of model 2 (R^2^: 0.9692 > 0.9642). The six BAF values obtained from cultivars grown in Jiangxi soil were closer to their predicted values than were those of the cultivars grown in Shaanxi soil, which indicated that the prediction models were better suited for wheat cultivars in acidic soil (Fig 3A and 3B). In a word, the good simulation of BAFs for six wheat varieties in two types of soil with opposite characteristics indicates that prediction models 1 and 2, which were developed using cultivar Xiaoyan 22, can be used to accurately predict Pb accumulation in the grain of other wheat varieties via cross-species extrapolation. There was no significant difference between the Pb-BAF of grain subjected to low and high Pb treatment; moreover, no significant difference existed between the two prediction models in different soil Pb levels. The current work was conducted in parallel with studies assessing prediction models of Pb transfer from soil to carrot (n = 21) [35] and corn (n = 17) [22] using the soil Pb concentrations used in our study.

**Table 3:** Intrinsic sensitivity (*k*) for non-modeled wheat cultivars extrapolated from the Xiaoyan 22-based model.

Prediction model	Intrinsic sensitivity (k)					
	Zhengmai 9023	Xumai 30	Wanmai 52	Shixin 618	Jimai 22	Hengmai 5229
Model 1	-1.58	-1.67	-1.74	-1.58	-1.58	-1.51
Model 2	-1.45	-1.54	-1.61	-1.45	-1.38	-1.38

### Normalization of BAF in different wheat varieties

It is inappropriate to directly apply short- or long-term toxicological data from different sources to develop environmental threshold-related or evaluation-related calculations, because the physical chemistry of the test medium, whether water or soil (water: pH, Ca^2+^, and Mg^2+^; soil: pH, OC, etc.), influences the predictions obtained from the input parameters (EC_50_, LC_50_, BAF, etc.) [12]. Accordingly, chronic ecotoxicity data (such as BAF) are required to normalize prediction models for the physico-chemistry of specific soils, which removes the effects of various soil properties on heavy metal accumulation from the model. For example, the Cd-BAFs of eight wheat varieties were normalized to a series of specific soil properties (pH: 5–9; OC: 5–25 g·kg^-1^) and used to modify a prediction model of Cd transfer from soil to grain in 18 Chinese soils [10]. Moreover, model normalization favors development of soil environmental quality criteria values for risk assessment through cumulative distribution functions using SSD methodology [21].

**Fig 4:**
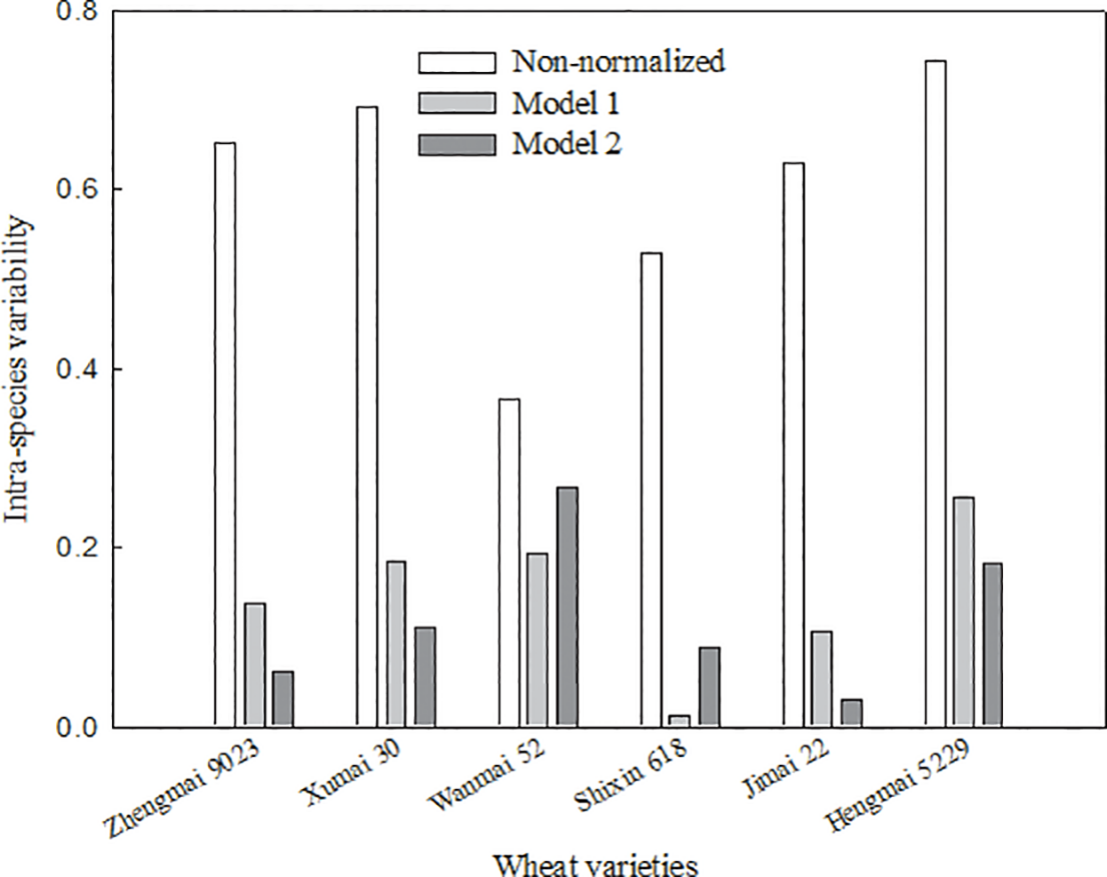
Intra-species variability in the Pb-BAFs of six wheat varieties before and after normalization.

The intra-species variability calculated by Eq 3 (before normalization) for each non-modeled wheat cultivar was higher than the corresponding value calculated following normalization (Fig 4). The average reduction of intra-species variability of the six varieties was 4.05 and 4.87 times for models 1 and 2, respectively, which demonstrated that the prediction models were effective in reducing the influence of soil properties on toxicological data (BAF). As in a previous study using corn cultivars, reduction of the intra-species variability in Pb uptake of the six non-modeled wheat cultivars was found when Pb-BAF was normalized to soil pH and OC [22]. Moreover, cross-species extrapolation of prediction models for Cd transfer from soil to corn grain also demonstrate that normalization is essential to reduce intra-species variability in predicted Cd transfer by corn varieties [21].

### Model validation using published data

**Fig 5:**
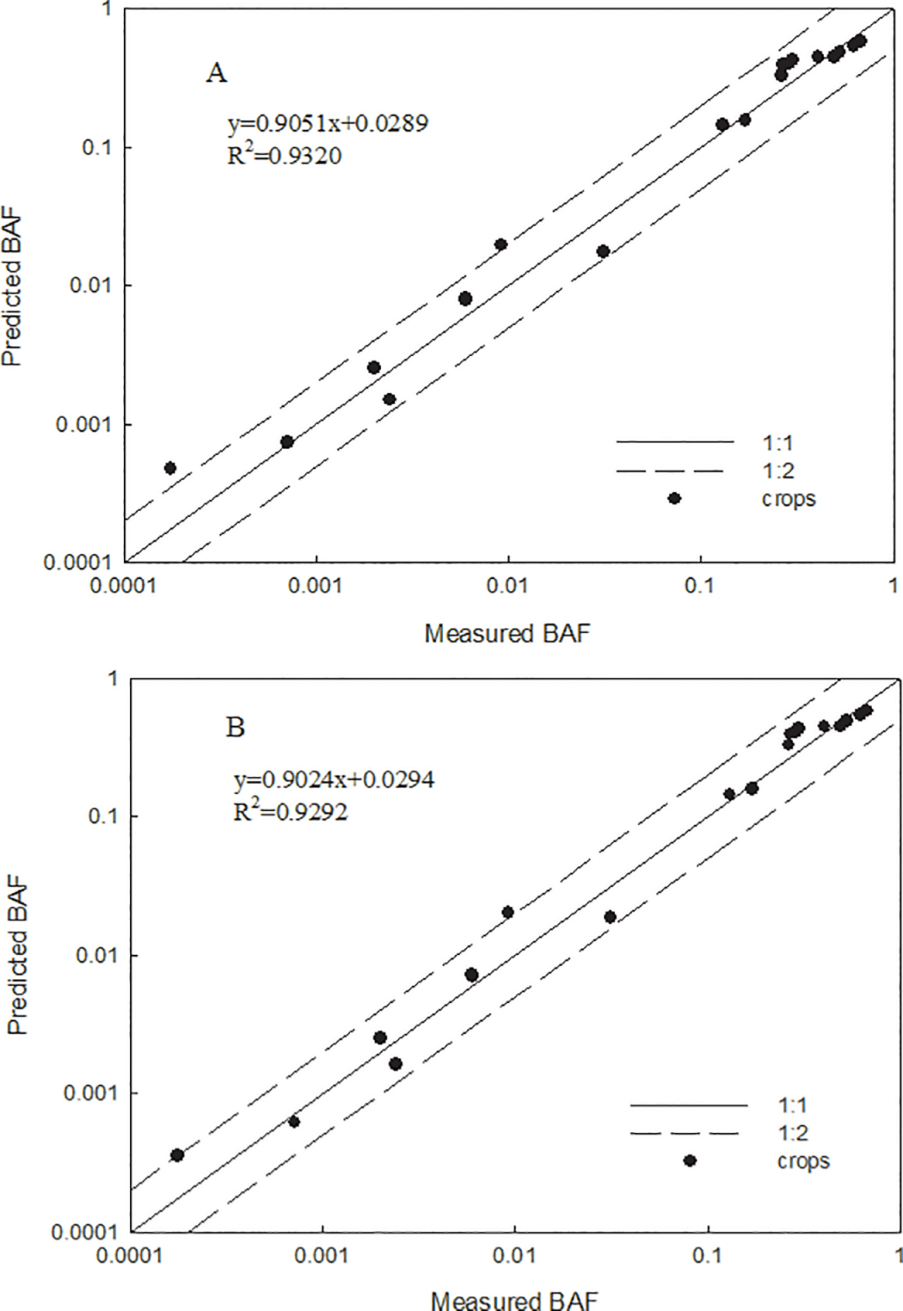
Relationship between measured and predicted BAFs for non-modeled crops. (the solid line indicates a perfect correlation; the dashed lines represent a 2-fold prediction interval; A, model 1; B, model 2).

**Fig 6:**
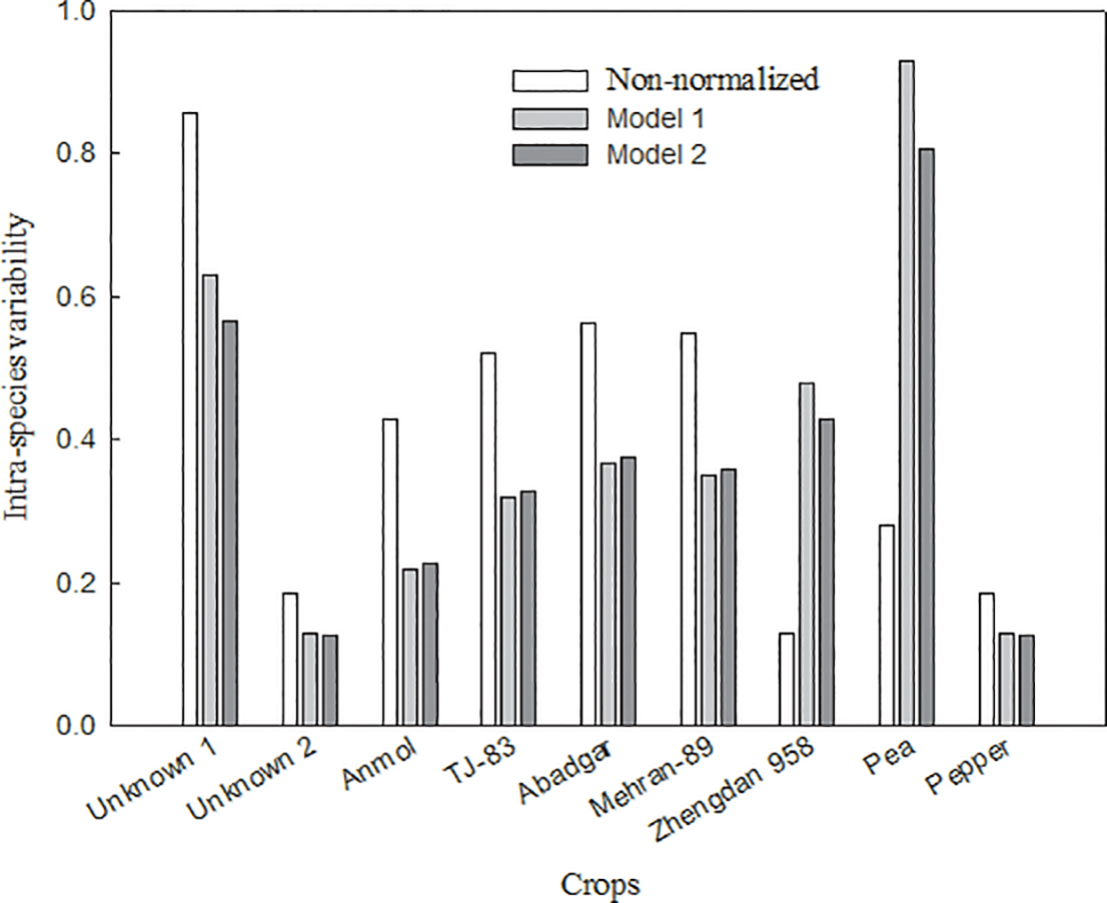
Intra-species variability in the Pb-BAFs of non-modeled crops before and after normalization.

In order to estimate the applicability of the prediction model 1, 2 developed using cultivar Xiaoyan 22, we calibrated the model for published datasets [22, 26, 36–38] by optimizing the BAF (S2 Table) and measured the differences between predicted and measured BAFs as an indicator of accuracy. To this end, normalization of the Pb-BAFs of non-modeled crops including six wheat cultivars was conducted using the prediction models described above (Table 2). However, unavoidably, the properties of the soils (pH: 4.12–8.67; OC: 10.21–356 g·kg^-1^) used in the referenced studies differed with those of the soils used in our study (Jiangxi and Shaanxi soil). The predicted BAFs were within 2-fold of the measured BAFs for crops in various soils. The predictive accuracy of model 1 for crops from the literature was slightly better than that of model 2 (R^2^: 0.9320 > 0.9292) (Fig 5A and 5B). Moreover, the average intra-species variability for most crops was reduced 1.16 and 1.23 times by model 1 and model 2, respectively, following normalization. An exception to this reduction in variability was Zhengdan 958, perhaps because of similar BAFs among types of soil (Fig 6). These results verified that the prediction models developed in this paper using wheat cultivar Xiaoyan 22 were capable of accurately predicting Pb toxicity for related wheat species from different sources and can be extrapolated to other crops. Therefore, normalization of ecotoxicity data and cross-species extrapolation are effective strategies for accurate prediction of metal transfer from soil to plants.

The strategy of developing prediction models of Pb phytotoxicity for untested wheat species or some other crops based on previously modeled species via extrapolation should spur the creation of a database containing important data regarding Pb toxicity, which will be useful to farmers, researchers, and policy makers. The prediction model provided herein should help policy makers improve soil-specific site risk assessments regarding Pb and establish accurate estimations of threats to food safety. However, additional field experiments should be conducted to further assess the feasibility of the prediction model reported in this study.

## Conclusions

Experiments in various soil types and wheat varieties indicated that Pb transfer from soil to wheat was positively associated with soil pH and OC. The Pb-BAFs of wheat grain grown in 17 types of soil with different soil Pb levels were well predicted by prediction models 1 and 2. There was no significant difference between the two models developed using wheat cultivar Xiaoyan 22, both of which can be used to predict the Pb-BAF of non-modeled wheat species and other crops, in which the predictive accuracy of model 1 was slightly better than that of model 2. Finally, this work provides information that could improve management of heavy metals in soil and public food safety.

## Supporting Information

S1 Fig Relationship between measured and predicted BAF_b_ for six wheat varieties in Shaanxi and Jiangxi soils (A, model 1; B, model 2).
(TIF)

S2 Fig Intra-species variability in the Pb-BAF_b_ of non-modeled crops before and after normalization. (TIF)

S1 Table Quality control results (mg·kg^-1^) obtained in the analysis of reference material. (DOC)

S2 Table Soil properties and BAF values for Pb from references. (DOC)

S3 Table Effects of bioavailability (0.05 M EDTA extractant) and total soil Pb on bioaccumulation (n = 17). (DOC)

S4 Table Prediction models of BAF_b_ based on the bioavailability of Pb in soil. (DOC)
