# Supplementary figures and images for: Cross-Species Extrapolation of Models for Predicting Lead Transfer from Soil to Wheat Grain
Source: PLoS One. 2016 Aug 12;11(8):e0160552. doi: 10.1371/journal.pone.0160552 (PMC4982616; doi:10.1371/journal.pone.0160552)

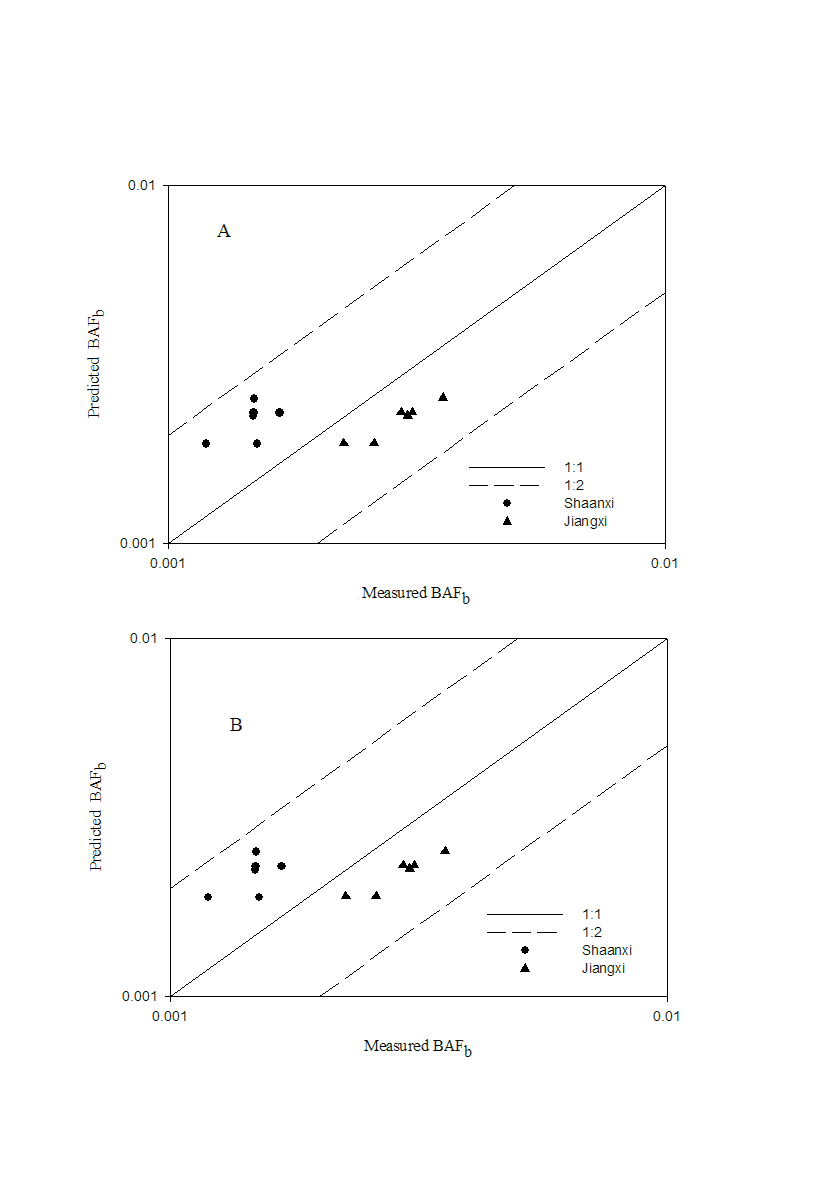

Supplement: S1 Fig — Relationship between measured and predicted BAFb for six wheat varieties in Shaanxi and Jiangxi soils (A, model 1; B, model 2). (TIF) [file pone.0160552.s001.TIF]

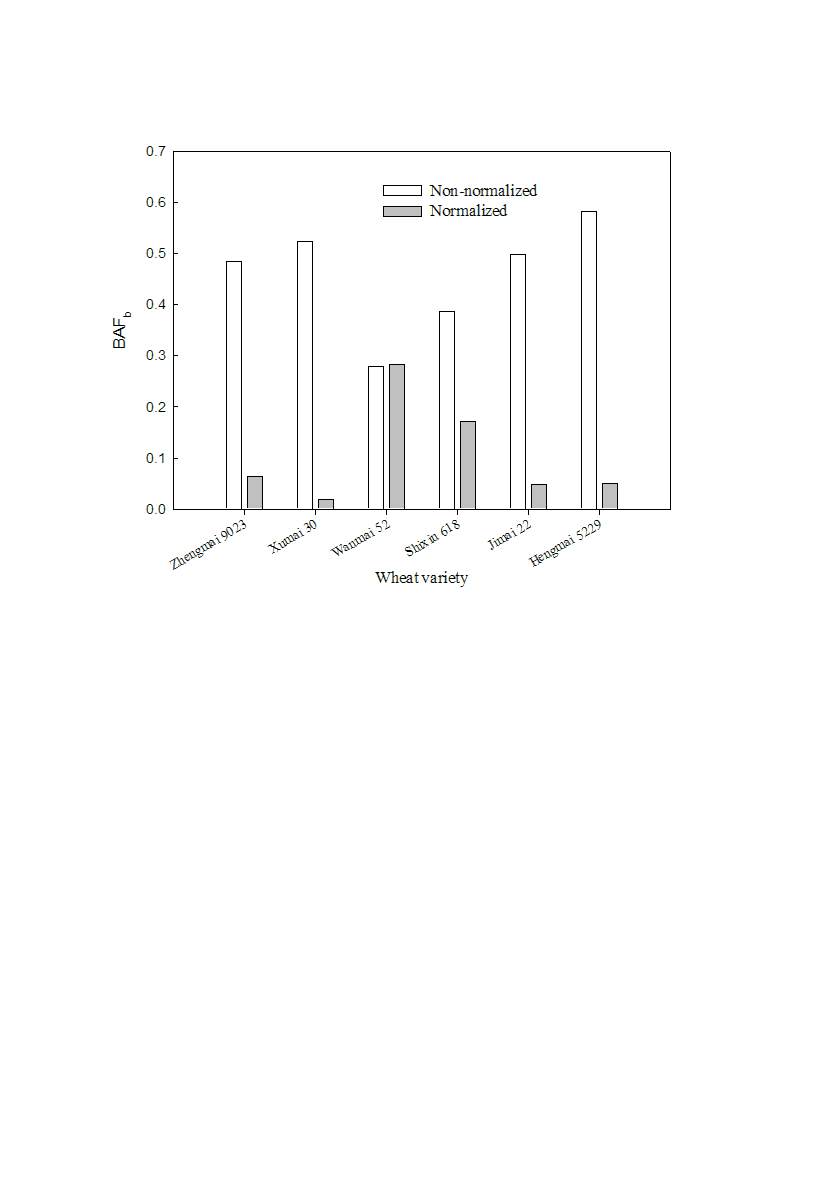

Supplement: S2 Fig — (TIF) [file pone.0160552.s002.TIF]
