# Supplementary material for: Cross-Species Extrapolation of Models for Predicting Lead Transfer from Soil to Wheat Grain
Source: PLoS One. 2016 Aug 12;11(8):e0160552. doi: 10.1371/journal.pone.0160552 (PMC4982616; doi:10.1371/journal.pone.0160552)
Supplement: S1 Table — (DOC) [file pone.0160552.s003.doc]

**Supporting information**

S1 Table. Quality control results (mg·kg^-1^) obtained in the analysis of reference material

| Material | NO. | Certified (mg·kg^-1^) |  |  | Experimental (mg·kg^-1^) | |  |
| --- | --- | --- | --- | --- | --- | --- | --- |
|  |  | Reference value |  | Mean | Median | Standard deviation | Coefficient of variation (%) |
| Wheat | GBW 10011 | 0.065±0.024 |  | 0.070 | 0.070 | 0.010 | 14.3 |
| Soil 1 (latosols) | Gss-7 | 14±4 |  | 15.76 | 15.29 | 2.32 | 14.7 |
| Soil 2 (loess) | Gss-8 | 21±3 |  | 24.23 | 23.53 | 3.13 | 12.9 |
