# Supplementary material for: Cross-Species Extrapolation of Models for Predicting Lead Transfer from Soil to Wheat Grain
Source: PLoS One. 2016 Aug 12;11(8):e0160552. doi: 10.1371/journal.pone.0160552 (PMC4982616; doi:10.1371/journal.pone.0160552)
Supplement: S2 Table — (DOC) [file pone.0160552.s004.doc]

**Supporting information**

S2 Table. Soil properties and BAF values for Pb from references

| Soil number | species | Cultivar | pH | OC(g·kg^-1^) | BAF | Reference |
| --- | --- | --- | --- | --- | --- | --- |
| 1 | wheat | Unknown 1 | 5.7 | 19 | 0.0007 |  |
| 2 | wheat | Unknown 1 | 7.3 | 19 | 0.0002 |  |
| 3 | wheat | Unknown 2 | 8.52 | 21.4 | 0.1699 |  |
| 4 | wheat | Unknown 2 | 8.67 | 22.4 | 0.1305 |  |
| 5 | wheat | Anmol | 7.53 | 257 | 0.4930 |  |
| 6 | wheat | Anmol | 7.7 | 356 | 0.2640 |  |
| 7 | wheat | TJ-83 | 7.53 | 257 | 0.6250 |  |
| 8 | wheat | TJ-83 | 7.7 | 356 | 0.2890 |  |
| 9 | wheat | Abadgar | 7.53 | 257 | 0.6250 |  |
| 10 | wheat | Abadgar | 7.7 | 356 | 0.2690 |  |
| 11 | wheat | Mehran-89 | 7.53 | 257 | 0.6770 |  |
| 12 | wheat | Mehran-89 | 7.7 | 356 | 0.2990 |  |
| 13 | corn | Zhengdan 958 | 6.3 | 10.21 | 0.0020 |  |
| 14 | corn | Zhengdan 958 | 5.9 | 22.39 | 0.0024 |  |
| 15 | pea | Unknown 3 | 4.12 | 26.4 | 0.0312 |  |
| 16 | pea | Unknown 3 | 4.54 | 19.7 | 0.0092 |  |
| 17 | pea | Unknown 3 | 6.69 | 28.5 | 0.0060 |  |
| 18 | pea | Unknown 3 | 6.84 | 28.2 | 0.0060 |  |
| 19 | Pepper (leaves) | Unknown 4 | 8.52 | 21.4 | 0.5274 |  |
| 20 | Pepper (leaves) | Unknown 4 | 8.67 | 22.4 | 0.4051 |  |
