# Supplementary material for: Cross-Species Extrapolation of Models for Predicting Lead Transfer from Soil to Wheat Grain
Source: PLoS One. 2016 Aug 12;11(8):e0160552. doi: 10.1371/journal.pone.0160552 (PMC4982616; doi:10.1371/journal.pone.0160552)
Supplement: S3 Table — (DOC) [file pone.0160552.s005.doc]

**Supporting information**

S3 Table. Effects of bioavailability (0.05 M EDTA extractant) and total soil Pb on bioaccumulation (n = 17)

|  | C_plant_/C_soil_ |  |
| --- | --- | --- |
|  | Low Pb | High Pb |
| C_plant_/C_b_ | 0.918^**^ | 0.749^**^ |

Note: ** highly significantly correlation, C_b_ is the bioavailability of Pb in soil, C_soil_ is the total soil Pb, the control is omitted because some C_b_ of soil sites are lower than the detection limits.
