# Supplementary material for: Cross-Species Extrapolation of Models for Predicting Lead Transfer from Soil to Wheat Grain
Source: PLoS One. 2016 Aug 12;11(8):e0160552. doi: 10.1371/journal.pone.0160552 (PMC4982616; doi:10.1371/journal.pone.0160552)
Supplement: S4 Table — (DOC) [file pone.0160552.s006.doc]

**Supporting information**

S4 Table. Prediction models of BAF_b_ based on the bioavailability of Pb in soil

| Model NO. | Treatment | Prediction models | R^2^ | *p* | n |
| --- | --- | --- | --- | --- | --- |
| Model 1 | Low Pb | Log BAF_b_=-0.001pH-0.004Log OC+0.012 | 0.68 | <0.001 | 17 |
| Model 2 | High Pb | Log BAF_b_=-0.001pH-0.007Log OC+0.015 | 0.67 | <0.001 | 15 |
| Note：model 2 removed NO. 12 and 13 soil sites, BAF_b_=C_plant_/C_b_ | | |  |  |  |
